# Green Starch Modification Using Citric Acid: Quinoa, Chickpea, and Cassava Starches

**DOI:** 10.3390/foods14020164

**Published:** 2025-01-08

**Authors:** Disala Menuwara Arachchi, Anthony Halim, Gbemisola Fadimu, Asgar Farahnaky, Mahsa Majzoobi

**Affiliations:** Biosciences and Food Technology, RMIT University, Bundoora West Campus, Plenty Road, Melbourne, VIC 3083, Australias3909645@student.rmit.edu.au (A.H.); fadimugbemisola@gmail.com (G.F.)

**Keywords:** starch citrate, natural modification of starch, resistant starch, dietary fibre, quinoa starch, underutilised crops, pulse starch

## Abstract

Dietary fibre deficiency has been associated with various global health challenges. Starch, as a main component of many staple foods, is typically very low in fibre content. The primary aim of this research was to increase the dietary fibre and alter the physicochemical properties of some common and emerging starches (cassava, quinoa, and chickpea starch) using eco-friendly modifications. Citric acid, a safe, natural, and environmentally friendly cross-linking agent, was employed for this purpose. Starch samples were treated with 30% citric acid and dry-heated at 130 °C for 5 h. This process resulted in relatively high degrees of substitution: 0.124 for cassava, 0.117 for quinoa, and 0.112 for chickpea starches. The modification successfully produced rich sources of dietary fibre suitable for food applications. It also reduced water interactions, pasting properties, and crystallinity. The highest reduction in swelling power and solubility was observed in quinoa starch (−67.34% and −82.10%, respectively), while the lowest values were obtained for cassava starch (−35.39% and −44.22%). All starches retained their granular integrity; however, they lost birefringence and Maltese crosses and showed some erosions on the granule surfaces. The citrate starches produced in this research offer thermally stable starch suitable for various food applications.

## 1. Introduction

Noncommunicable diseases (NCDs) such as diabetes, cardiovascular diseases, cancers, and obesity are highly prevalent diseases globally that are largely attributed to unhealthy dietary habits [1]. Dietary fibre is a well-known food component that is essential in a healthy diet and in the prevention of most of the NCDs. The lack of dietary fibre intake has a strong link to the rising prevalence of diabetes and obesity [2]. The main dietary fibre related to starch is “resistant starch”, a type of starch that resists digestion in the human gut. Resistant starch occurs naturally in small amounts (often less than 5%), but it can be significantly increased through modification techniques [1]. Incorporating resistant starch into food formulations is a strategic approach employed by food scientists to reduce the glycemic index and glycemic load of these products, thereby contributing to better health outcomes and the prevention of highly prevalent NCDs [1,3].

Resistant starch is classified into five different types based on the structure and method of formation, including RS1 (the naturally occurring resistant starch), RS2 (the intact and gelatinised starch), RS3 (retrograded starch), RS4 (produced by chemical modification of starch), and RS5 (amylose–lipid complex) [4]. Cross-linking is a promising technique to produce dietary fibre, specifically resistant starch type 4 (RS4). RS4 starch is highly valued in food products due to its lower digestibility compared to native starch and its health benefits, which are similar to those of other dietary fibres [3,5]. Additionally, cross-linking is a widely used chemical method for modifying starch to improve starch resistance to retrogradation, enhance freeze–thaw stability, and increase stability during cooking, shearing, and processing under various temperatures and pH conditions. Phosphorus oxychloride, epichlorohydrin, sodium tripolyphosphate, and sodium trimetaphosphate are common cross-linking agents. However, the main concern with the cross-linking process is the hazardous and toxic nature of the chemicals used and their environmental impact. Therefore, the amount of chemical cross-linker used in food applications must adhere to the guidelines set by the FDA and other global food regulatory bodies. To address these issues, natural, non-toxic, and generally-recognised-as-safe (GRAS) cross-linking agents, such as butanetetracarboxylic acid, maleic acid, and citric acid, have been introduced [5,6].

Citric acid, in particular, is considered safe for both the environment and human health. The JECFA does not set specific limitations on using starch citrate in foods. Naturally occurring in many plants and food products, citric acid is a highly soluble organic acid used as a common food ingredient. With its three carboxyl groups, citric acid serves as a cross-linking and esterifying agent in starch modification, making it a preferred natural and reliable food additive [7,8].

**Figure 1:**
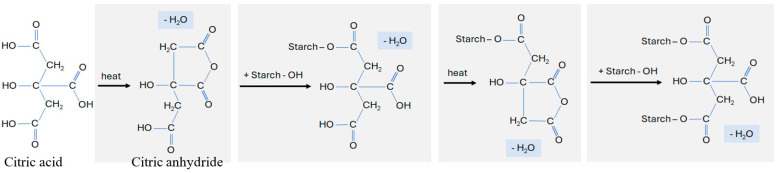
Cross-linking effect of citric acid on starch at elevated temperatures.

The effect of citric acid on starch is strongly influenced by the processing conditions. When processing temperatures are kept below the gelatinisation temperature, starch hydrolysis may occur. However, at elevated temperatures (above 100 °C) and in dry conditions, as used in this research, citric acid acts as a cross-linking agent. The cross-linking mechanism between starch and citric acid is depicted in Figure 1. When citric acid is dry-heated, a citric anhydride intermediate forms, serving as the base mechanism responsible for the development of cross-links with starch. Esterification of the -OH functional groups of the starch with the cyclic anhydride intermediate leads to the formation of new carboxylic acid units. These units exhibit the ability to form new intramolecular anhydride moieties with neighbouring carboxylic acid units, preferentially at the least-hindered C-6 position of the glucose, to create ester groups. This process forms inter- and intramolecular bonds at random locations within the starch granules through cross-linking. Conversely, the evaporation of water, the initial step in cross-linking with citric acid, is facilitated by the hydrophobic nature of starch [5,9,10].

Citric acid cross-linking significantly alters the granular morphology and molecular structure of starch, leading to changes in functional properties such as digestibility, water interaction, and viscosity. The magnitude of these changes is influenced by the type of starch and processing conditions, including moisture content, temperature, duration, pH, and acid concentration [7,8].

Citrate modification can significantly alter the morphology and structure of starch granules, leading to changes in their functional properties [11]. Similar to other starch cross-linkers, citrate starch, especially when used at higher levels (>20%), can significantly decrease starch digestibility and the glycaemic index and increase the resistant starch content (RS4). Various values of resistant starch, ranging between 20 and 80%, have been reported for citrate starch depending on the modification conditions and starch type [12,13,14]. Thus, citrate modification can be considered as a green strategy to enhance resistant starch content (known as a dietary fibre). 

The production of citrate starch is a mild, convenient, and eco-friendly process that has been successfully applied to cross-link various types of starches, including those from wheat, potatoes, lentils, and cassava. However, there is a scarcity of information regarding its effects on underutilised starches such as quinoa starch and emerging pulse starches like chickpea starch [15]. These novel starches are in the spotlight with regard to the growing interest in diversifying the human diet with more environmentally friendly crops. Additionally, these starches often show unique functional properties such as superior emulsifying properties and being gluten-free, making them a valuable natural ingredient [16,17]. 

The primary goal of this research was to increase the fibre content and create new functionalities in cassava, quinoa, and chickpea starch by citric acid cross-linking modification, employing a reliable, plant-based, and eco-friendly modification strategy for novel food applications. This research addresses the global demand for novel sources of health-promoting foods derived from sustainable and environmentally friendly sources. The novelty of this research lies in comparing emerging starches like quinoa and chickpea with mainstream cassava starch. These emerging starches are gaining attention due to their value in diversifying diets and promoting eco-friendly, sustainable food options. This study is particularly valuable for the industry, as we introduce a green, semi-dry, and efficient method to form dietary fibre. 

## 2. Materials and Methods

### 2.1. Materials

Quinoa flour was obtained from Bob’s Red Mill Natural Foods, Inc. (13521 SE Phaesant Court, Portland, OR, USA). Chickpea starch was obtained from Hela Spice Australia Pty Ltd. (132 Woodlands Drive, Braeside, Victoria, Australia, batch number—Lab0604). Cassava starch was obtained from Ingredion ANZ Pty Ltd., North Ryde, Australia. The Megazyme Kit for measuring the total dietary fibre content was purchased from Neogen Australia (Bundamba Australia). All other chemicals were of analytical grade, and obtained from Sigma Aldrich, North Ryde, Australia.

### 2.2. Extraction of Quinoa Starch

Quinoa starch was produced from quinoa flour using the alkaline steeping (0.25% NaOH) method [18]. Briefly, 100 g of quinoa flour was mixed well with 600 mL 0.25% NaOH in a jar at room temperature, and then covered and stored at 4 °C for 24 h. The top brown liquid was carefully discarded, and the pellet was recovered. The pellet was passed through sieves with mesh sizes of 55, 110, and 150 in order and the final filtrate was collected, washed five times with distilled water, followed by centrifugation at 3000× *g* for 10 min until a clear supernatant was obtained. The collected pellet was dried at 40 °C overnight, milled and sieved to obtain a particle size of 130 microns, and stored in an airtight container at ambient temperature.

### 2.3. Preparation of Citrate Starch

Citrate starch was prepared by drying at a high temperature based on the method described by Mei et al. (2015), with slight modifications [13]. Citric acid (30% based on the starch’s dry weight) was dissolved in 30 mL of distilled water and the pH was adjusted to 3.5. The solution was diluted to a final volume of 60 mL and manually mixed thoroughly with 50 g of native starch. The mixture was kept at room temperature for 6 h in an aluminium tray and then dried at 50 °C for 24 h in a drying oven. The dried mixture was ground using a coffee grinder and heated at 130 °C for 5 h. The dried mixture was washed three times using distilled water. Then, it was dried overnight at 45 °C using a drying oven, ground, and stored in airtight containers.

### 2.4. Degree of Substitution (DS)

To determine the amount of citric acid esterified to the starch, a reaction was carried out between citric acid and Cu^2^⁺, resulting in the formation of a stable complex during titration with a copper sulphate solution [13]. The DS was calculated based on the average number of substituent groups per anhydroglucose unit. A starch sample (450 mg) was then dispersed in 2 mL of deionised water and dissolved in 50 mL of 1 M KOH. The solution was heated in a water bath set at 90 °C for 10 min. After cooling the solution to 25 °C, its pH was adjusted to 8.5 using 5 M acetic acid. The solution was then added to 25 mL of sodium borate buffer (pH 8.5) containing an indicator (0.3 g of a Murexide sulphate mixture in a 1:500 weight ratio) and diluted to 300 mL with deionised water. The solution was titrated with 0.05 M copper sulphate solution until the red-violet colour of the indicator disappeared. The DS was then calculated using Equation (1):

(1) $$DS=\frac{\left(162\times W\right)}{\left(100\times M\right)}-\left(M-1\right)\times W$$

where W (% by weight of substituent) = [bound citrate (g)/sample (g) − bound citrate (g)] − 100, and M = molecular weight of the citric acid substituent, which was 175.1 [13]. 

### 2.5. Determination of Total Dietary Fibre Content

The total dietary fibre content of the native and citrate starches was measured using the Megazyme Total Dietary Fibre Kit (Megazyme, Irishtown, Ireland) by following the procedure in [5].

### 2.6. Morphological Properties of Starch Granules Using Polarised Light Microscopy

The polarised light microscopic images of the native and citrate starches were taken at 40× using the BX53-Olympus polarising light microscope. Three images were taken of each sample without and with the polariser [8].

### 2.7. Morphological Properties of Starch Granules Using Scanning Electron Microscopy (SEM)

The morphological features of the samples were examined using SEM (Quanta 200, FEI, OR, Salem, MA, USA). A dry, finely ground sample was positioned on 12.6 mm aluminium slabs and coated with 5 nm of iridium. Surface micrographs were acquired in high-vacuum mode, employing an accelerated electron beam of 10 kV, spot size of 4, and various magnifications [8]. 

### 2.8. Solubility and Swelling Power

Starch (0.2 g, dry basis) was dissolved in 20 mL distilled water in a centrifuge tube and kept in a water bath at 85° for 30 min, with intermittent mixing every 5 min using a vortex mixture. The tube was then rapidly cooled to room temperature by holding it to running tap water. After that, the tube was centrifuged at 3000× *g* for 15 min. The supernatant was carefully decanted and dried at 105 °C. The dried supernatant and the precipitate were weighed separately. Experiments were conducted in triplicate and the average was considered. Equations (2) and (3) were used to measure the solubility and swelling power, respectively [19].

Solubility (%) = (Weight of the dried supernatant/Dry weight of the starch) × 100(2)

Swelling power (g/g) = Weight of the precipitate in the tube/Dry weight of the starch(3)

### 2.9. Determination of Particle Size Distribution

The particle size analysis was conducted using a laser diffraction instrument (Mastersizer 3000 ATA, London, UK) according to the method used by Kumar et al. (2022), with some modifications. Native and citrate starches were dispersed in distilled water at room temperature and thoroughly mixed using a magnetic stirrer at 550 rpm for 5 min to form a 1% (*w*/*v*) homogenic dispersion. The measurements were conducted five times with 5–15% obscuration and a residual percentage below 2% [19].

### 2.10. Colour

Colour parameters of native and citrate starches were determined using a handheld Chroma meter (Minolta CR-400, Osaka, Japan). The calibration was conducted using a standard whiteboard. The total colour change (ΔE) and the whiteness index (WI) were calculated using Equations (4) and (5) [11].

ΔE = [(L*_native_ − L*)^2^ + (a*_native_ − a*)^2^ + (b*_native_ − b*)^2^]^½^ (4)

WI = 100 − [(100 − L*)^2^ + (a*)^2^ + (b*)^2^]^½^ (5)

where L*_native_ = Lightness of native starch, a*_native_ = Redness of native starch, b*_native_ = Yellowness of native starch, L* = Lightness of citrate starch, a* = Redness of citrate starch, and b* = Yellowness of citrate starch. 

### 2.11. FT-IR Spectroscopy

All infrared spectra were obtained using an FT-IR instrument (FT-IR-GladiATR-PIKE Technologies, Madison, WI, USA) according to the method used by Mei et al. (2015), with some modifications [13]. A spectral resolution of 4 cm^−1^ was used, and 64 scans of each sample were recorded and analysed. The spectra were obtained in the range of 400–4000 cm^−1^. The spectra were baseline-corrected and deconvoluted with Gamma 0, smoothing length 95%, and a Bessel filter. The absorbance ratio (R-value) of 1047 cm^−1^/1022 cm^−1^ was taken using the PerkinElmer Spectrum IR software—version 10.7.2.

### 2.12. Thermal Properties

The thermal properties of the starch were determined using DSC-Q2000-TA instruments (TA Instruments, Rydalmere, Australia) according to the method used by Mei et al. (2015). Starch (5 mg, dry basis) was mixed with 10 µL of distilled water in a DSC pan and equilibrated for 24 h at 4 °C. The pan was heated from 20 °C to 130 °C at a rate of 10 °C/minute. An empty pan was used as a reference. The Thermal Analysis Simultaneous DSC Universal Analysis software (version 4.5A) was used to calculate the onset, peak, conclusion temperatures, and gelatinisation enthalpy [19].

### 2.13. X-Ray Diffraction (XRD)

An X-ray Diffractometer (D4 Endeavour, Bruker, VIC, Australia) with Cu Kα radiation was used to measure the XRD pattern. The measurements were taken according to the method used by Mei et al. (2015), with some slight modifications [8]. The radiation was operated at 40 kV and 35 mA. The starch samples were dried in a desiccator at room temperature for three days. The scanning was conducted at a diffraction angle (2θ) of 6–80. The diffractograms were smoothed and baseline-corrected. The analysis was conducted using the Origin Pro software (version 9.8.5.212).

### 2.14. Pasting Properties

The pasting properties of starch were determined by the Rapid Visco Analyser (RVA 4500-Perten instruments, Sydney, Australia). Native or citrate starch (3.0 g, dry weight) was mixed with distilled water based on the moisture content of the starch in RVA sample canisters. The standard RVA program was used, where the samples were held at 50 °C for 1 min, heated to 95 °C with a heating rate of 11 °C/minute, cooled to 50 °C with a heating rate of 11 °C/minute, and then held at 50 °C before terminating the cycle. Pasting parameters including peak, breakdown, setback, and trough and final viscosities were obtained from the RVA graph [19]. 

### 2.15. Gel Texture

After the RVA test, the canisters containing starch paste were covered with parafilm and stored at 4 °C for 24 h to form a gel. Then, the textural properties of the gels were examined using a Texture Analyser (TA.XT Plus, Stable Microsystems, Birmingham, UK), using a double compression test with a cylindrical probe (SMS P/50) [18]. The samples were compressed twice at a speed of 5 mm/s with a strain of 25% and a trigger force of 4 g and a resting time of 5 s. A force–time graph was generated, and the hardness, cohesiveness, and gumminess of the samples were derived. 

### 2.16. Statistical Analysis

The results are expressed as the mean value ± standard deviation. The compilation of results and creation of graphs were conducted using Microsoft Excel (version-18.2306.10161.0) and data analysis was conducted by One-Way Analysis of Variance (ANOVA) and Tukey’s test. A value of *p* < 0.05 was considered significant.

## 3. Results and Discussion

### 3.1. DS of the Citrate Starch

The DS of a starch derivative is defined as the number of hydroxyl groups substituted per d-glucopyranosyl ring. Each ring contains three hydroxyl groups, so the maximum possible DS is three. However, the primary hydroxyl group at the C-6 position is more reactive than the secondary hydroxyl groups at the C-2 and C-3 positions due to steric hindrance [20]. The DS is influenced by various factors such as the source of starch, amylose and amylopectin content, starch composition, reactant concentration, and reaction time and temperature. Under similar modification conditions, starches with a higher DS are more reactive to the modification [5]. As shown in Table 1, the DSs of citrate cassava, quinoa, and chickpea starches produced using similar conditions were 0.124 ± 0.011, 0.117 ± 0.008, and 0.112 ± 0.007, respectively, which were not significantly different (*p* < 0.05). Different DS values were reported in the literature for citrate starches, ranging from 0.01 to 0.42 depending on the starch type, processing condition, and ratio of citric acid to starch [21]. For citrate cassava starch a DS value of 0.17 was obtained when treated with 30% citric acid [13]. Unlike chemically cross-linked starches, there is no set limitation for the use of citric acid in starch modification and citrate starches with various DSs can be produced depending on the application [22].

### 3.2. Total Dietary Fibre Content

The conversion of native starch into citrate starch resulted in a significant increase in the total dietary fibre content (Table 1) and a maximum of 95.53% was obtained for citrate chickpea starch. During citrate modification, citric acid is readily converted to its anhydrous derivative upon heating, enabling it to react with the hydroxyl groups of starch. It is well established that during the reaction of starch with citric acid, esterification begins with the formation of citrate starch, and further heating of the reaction medium promotes cross-linking reactions [5,9,10]. Citric acid modification can reduce the absorption of starch in the body. This occurs because the enzymatic digestion of starch is hindered by the formation of derivative groups, which interfere with the formation of enzyme–substrate complexes. In other terms, citrate starch is a type of resistant starch (RS4) and is categorised as a dietary fibre [1,4,13]. An increase in starch resistance to enzymatic reactions and a reduction in its digestibility have been reported for citrate-modified cassava, corn, sorghum, and rice starches [13,14,23]. However, it has been reported that the high level of citric acid (<30%) can have a negative effect on resistant starch formation since accumulation of citric acid can physically prevent further cross-linking of starch [13]. 

### 3.3. Colour Test

Colour is a sensory property associated directly with the quality and purity of starch. According to the results (Table 2), native starches showed no significant difference in lightness but exhibited significant differences in redness and yellowness values. These variations can be attributed to the intrinsic differences between the tested starches, including differences in their natural pigments and chemical composition. Citric acid modification had no significant impact on the lightness of the samples compared to their native counterparts but reduced the whiteness index (WI) and redness (a*) while it increased yellowness (b*). Notably, yellowness increased approximately threefold following citric acid modification. These colour changes resulted in significant differences in the ∆E values, with the largest difference observed being for the citrated quinoa. It has been reported that the lightness and yellowness of citrate rice starch decreased and increased, respectively, which agrees with the results of the present research [11]. The discolouration of starch during citric acid modification can be due to the dehydration of citric acid, which bestows a colour on the starch. In addition to this, starch undergoes partial degradation at higher temperatures, which also leads to discolouration [7]. This discolouration could be a disadvantage in the commercial applications of modified citrate starches, and thus further bleaching may be suggested.

### 3.4. Thermal Properties

**Figure 2:**
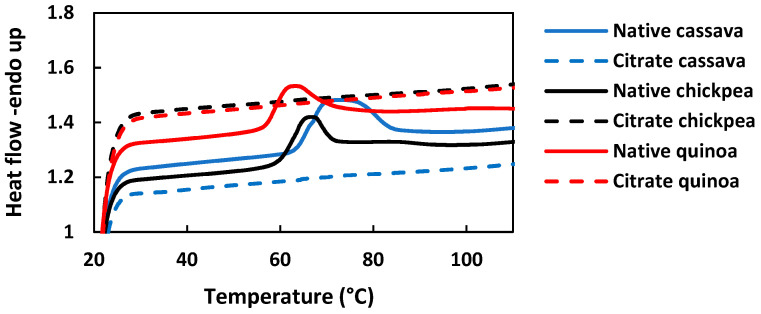
DSC thermograms of native and citrate starches.

The DSC thermograms of native and citrate starches are shown in Figure 2, while the gelatinisation parameters are shown in Table 3. All the gelatinisation parameters of the three native starches were significantly different due to their intrinsic differences in starch molecular structure and composition (*p* < 0.05). Native cassava showed the highest gelatinisation parameters, while native quinoa showed the lowest parameters except for the ∆H. There was no significant difference between the ∆H of native quinoa and native chickpea starches. The ∆H value is a representation of the number of double helices that were untangled and melted during the gelatinisation process [24]. The higher ∆H values of native starches are an indication that a high amount of thermal energy is required to melt the double helices of the starch granules [25]. These results align with the crystallinity index of native starches (Figure 2). The higher the crystallinity index, the higher the gelatinisation enthalpy. Since native cassava starch had the highest crystallinity, it needed the highest energy to untangle and melt the double helices.

The endothermic peak, which was present in native starches, was absent in all three citrate starches. Due to this, the gelatinisation parameters of any of the citrate starches could not be determined. This indicates that the cross-linking reaction caused by the addition of citric acid increased the number of amorphous regions of the starch granules, and thereby negatively affected the starch crystallinity [13]. According to these results, partial esterification can reduce the gelatinisation parameters of starch. Disappearance of the endothermic peak in the DSC thermogram was reported for 30% citrate cassava starch [13], for 48% and 60% citrate rice starch [11], and for 40% citrate wheat starch [26].

**Figure 3:**
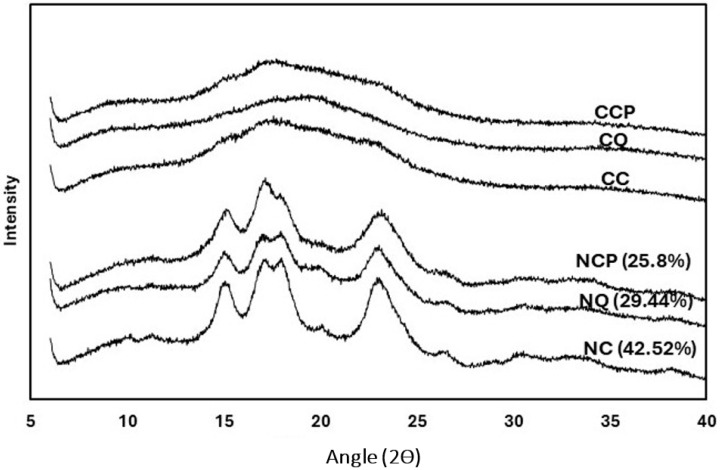
The X-ray diffraction patterns (smoothed and baseline-corrected) of native and citrate starches. NC = Native cassava starch; NQ = Native quinoa starch; NCP = Native chickpea starch; CQ = Citrate quinoa starch; CCP = Citrate chickpea starch; CC = Citrate cassava starch.

The low ∆H value of citrate starches, due to the absence of the endothermic peak, is strong evidence to prove that the cross-linking by CA has altered the chain packing, thereby reducing the crystallinity and increasing the amorphous nature of starch molecules by forming new covalent and hydrogen bonds. These results are supported by the XRD diffractograms (Figure 3).

### 3.5. Starch Crystallinity

The X-ray diffraction patterns of native and citrate starches are shown in Figure 3. Native quinoa and cassava starch showed a typical A-type pattern, with single peaks at 2θ of 15° and 23° and a doublet at 17° and 18°, consistent with previous reports [27]. Similarly, an A-type crystalline pattern for native cassava starch has been reported [13]. Native chickpea starch showed a C-type crystalline pattern, with strong peaks at 2θ of 15°, 17°, and 23° [17]. The highest Crystalline Index (CI) was observed in native cassava (42.52%), while the CI of native chickpea and native quinoa starches was 25.80% and 29.44%, respectively. The CI aligns with the ∆H values obtained through DSC thermograms (see Figure 1). The results in the present research showed a direct proportionality between these two values, where native cassava displayed the highest ∆H and CI and native chickpea showed the lowest ∆H and CI. These findings are correlated to the differences in the branching patterns of amylopectin in these starches, which are crucial in determining the type of unit packing and X-ray diffraction pattern [22].

**Figure 4:**
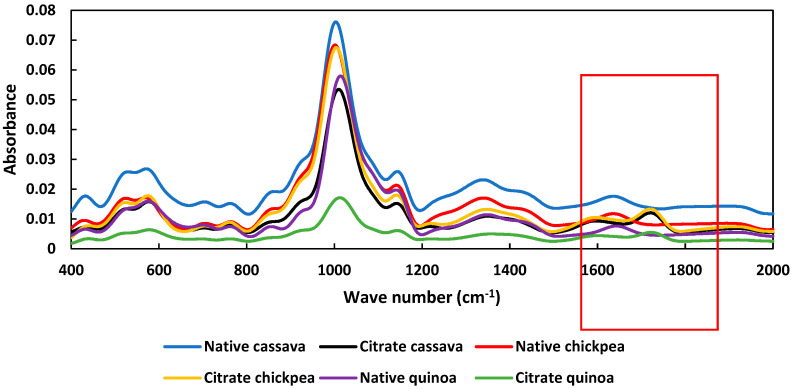
The deconvoluted FTIR absorbance spectra of native and citrate starches.

After citrate modification, the X-ray peaks disappeared in all three citrate starches, indicating an increase in the amorphous region and a decrease in the crystalline region of the citrate starches. When citric acid penetrates the starch granules, it can disrupt both amorphous and crystalline structures due to the concentrated citric acid solution. The substitution of citric acid groups on the starch chains forms a highly cross-linked starch, thereby limiting the mobility of the starch chains. Previous studies using various levels of citric acid and different types of starches have reported a decrease in starch crystallinity. The increase in the degree of substitution results in more destruction of the crystalline structure, as reported for citrate cassava and rice starches [11,13]. These observations are also confirmed by the DSC thermograms (Figure 2) and FT-IR spectra (Figure 4).

### 3.6. FT-IR Results

The FT-IR spectra of native and citrate starches are shown in Figure 4. The absorbance peaks at 857 cm^−1^ and 930 cm^−1^ were due to the C-H bending, while the absorbance peak at 1350 cm^−1^ corresponded to O-C-H, C-O-H and C-C-H bending [13]. According to Figure 4, in all three citrate starches a new peak appeared at 1724 cm^−1^, which was not observed in their native counterparts. This new peak represented the ester group from citric acid. This peak was related to the vibration of the C=O bond within the acetyl group. This confirms the successful esterification of citric acid. The appearance of a new peak at 1724 cm^−1^ for 30% citrate cassava starch has been reported [13]. The absorbance peak at 1637 cm^−1^, which was present in native starches, was absent in citrate starches. This peak might be due to the carbonyl groups present in native starches, possibly due to oxidation [9,13].

**Figure 5:**
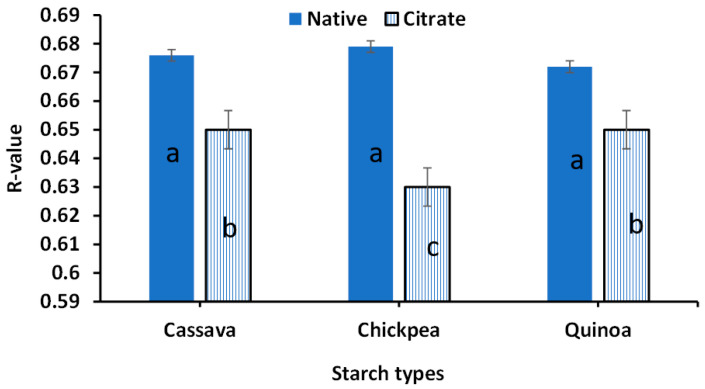
The R-value of native and citrate starches obtained from FTIR. Different letters on the columns show a significant statistical difference (*p* < 0.05).

Figure 5 gives the R-values, which represent the degree of order in starch granules. The absorbance band at 1047 cm^−1^ is associated with crystalline structures and the band at 1022 cm^−1^ is associated with amorphous structures [24]. It was evidenced that the R-value was lower in citrate starches compared to their native counterparts. This showed that crystallinity in citrate starches was lower than native starches. The lowest R-value was observed in citrate chickpeas (0.63), followed by citrated cassava and quinoa starches (~0.65).

### 3.7. Water Interactions

The solubility and swelling power of native and citrate starches are shown in Table 4. As compared to the native counterparts, citrate starches showed a significantly lower solubility and swelling power. The reduction in solubility of citrate cassava, chickpea, and quinoa was approximately 44%, 57%, and 82%, while the reduction in swelling power was 35%, 65%, and 67%, respectively. The highest reduction in solubility and swelling power was observed for quinoa starch, while the lowest reduction was found for cassava starch. The cross-linking of starch by citric acid changes the inter- and intra-connections between amylose and amylopectin and limits the swelling degree of starch granules [28]. It has been reported that citrate cassava and rice starches showed a lower solubility and swelling power than their native counterparts [11,13]. However, it has been indicated that the strengthening of bonds and the steric hindrance caused by the cross-linking restrict water penetration into the starch granules [8]. The reduction in solubility and swelling power strongly indicated successful citric acid modification and the production of cross-linked starch, suggesting enhanced thermal stability. Based on the outcome of the present research, it can be concluded that 30% citrate starch was highly stable in hot water and can be a potential candidate for canned foods [29].

### 3.8. Particle Size Distribution

The particle distribution information of native and citrate starches is given in Table 5. The cross-linking of native starches with citric acid significantly increased (*p* < 0.05) all the particle size parameters. The highest increase in diameter was observed for quinoa starch. The smaller fractions (D10) were significantly higher in citrate starches, which is similar to previous reports for citrate rice and tiger nut starches [8,11]. The increase in mean granule size after the cross-linking has also been reported for rice starch [11]. The span, which indicates the distribution width, was significantly increased (*p* < 0.05) after cross-linking due to the distributional shift towards the higher particle size [11]. This was reflected in the significantly increased D90 values. The volume-weighted mean diameter was significantly higher (*p* < 0.05) in citrate starches, lying in the range of 21.62–24.94 µm. This indicated a significant increase in the volume-weighted mean area. The increase in the particle size can be related to the agglomeration of the starch granules, which was also observed under a light microscope (see Figure 7). However, an opposite result was reported for tiger nut starch citrate, which was related to applied heat treatment and the penetration of citric acid during the reaction [30].

### 3.9. Pasting Properties

**Figure 6:**
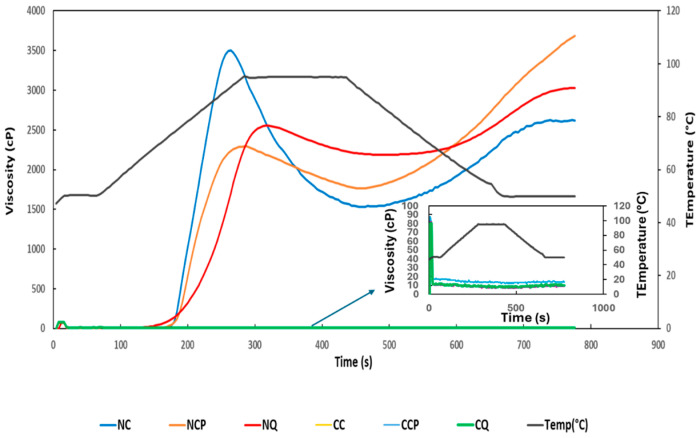
RVA graphs of native and citrate starches. NC = Native cassava starch; NCP = Native chickpea starch; NQ = Native quinoa; CC = Citrate cassava starch; CCP = Citrate chickpea starch; CQ = Citrate quinoa starch.

The RVA viscographs and pasting parameters of native and citrate starches are shown in Figure 6 and Table 6. There was a significant difference (*p* < 0.05) among the peak viscosity (PV) of the three native starches. PV shows the highest viscosity reached by starch during heating and mixing. Native cassava showed the highest PV, while native chickpea showed the lowest. Continuous mixing and heating after PV resulted in a drop in viscosity, measured as trough viscosity (TV) and setback viscosity (SV). TV and SV are indicators of starch paste stability during simultaneous heating and mixing. The higher values of TV and lower values of breakdown viscosity (BV) show a more stable starch paste [11]. Quinoa starch showed the highest TV, followed by chickpea and cassava starches. This indicates that the quinoa starch paste was more stable during simultaneous heating and mixing than the other two starches. This was also confirmed by the lower BV. The BV of native cassava was significantly higher than the others, indicating the higher sensitivity of cassava starch to mixing and heating. There was no significant difference in the SV of native cassava or quinoa starches. However, the SV of native chickpea starch was significantly higher than the other two (*p* < 0.05). The final viscosity (FV) indicates the viscosity during cooling (gelling process) and the tendency towards retrogradation. SV was calculated as the difference between FV and TV [24]. Native chickpea starch showed the highest FV, while cassava starch showed the lowest FV. The SV of the chickpea starch was the highest, followed by cassava and quinoa starches.

The pasting profiles of all three cross-linked citrate starches became flat when 30% citric acid was used, and all pasting parameters disappeared. The cross-linked starches were unable to develop viscosity due to the hindered water interactions, swelling and gelatinisation of starch granules (as explained in Section 3.7 and Section 3.8), and limited leaching of amylose during the heating and cooking stages owing to the high level of cross-linking [11]. Similar results have been reported for citrate cassava, sweet pea, lentil, rice, corn, and banana starches [8,11,13]. 

These observations indicate an improved thermal stability of citrate starches compared to their native counterparts due to the strengthening of the swollen starch granules by the formation of covalent bonds between the cross-linker and the starch granules and the changes caused by citric acid to the glycosidic linkages of amylopectin [7,13,31]. These also point out a lower tendency of the citrate starch granules to retrograde after cooling. The utilisation of citric acid as a cross-linking agent inhibits water penetration into starch granules, consequently diminishing water interaction, swelling, and gelling properties. These observations are a clear indication that 30% citrate starches can withstand mechanical shear and high temperatures. This behaviour is also supported by the reduced swelling power of all three citrate starches (Table 4). 

### 3.10. Textural Properties

**Figure 7:**
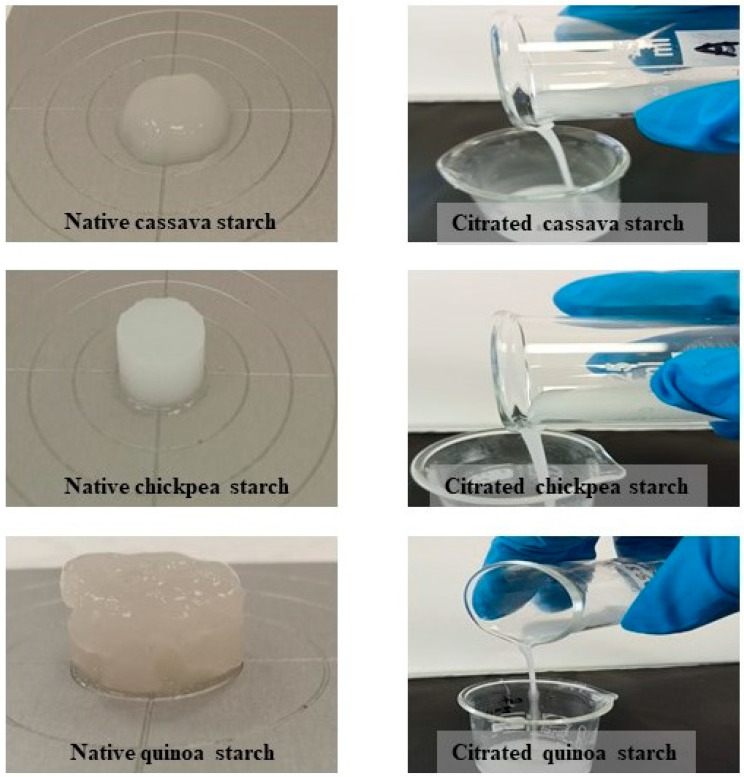
Gels of native cassava, chickpea, and quinoa starch gel and citrate cassava, citrate chickpea, and citrate quinoa starch samples after RVA pasting and cooling and overnight storage. A total of 12% starch was in water.

The samples obtained after the RVA analysis were cooled down and left overnight to form a gel for textural analysis. Figure 7 shows the appearance of the starch gels. Unlike their native counterparts, starches formed no gel after being subjected to a heating and cooling cycle. Similar results were reported for citrate corn and waxy corn starches heated at 100 °C for 30 min and then cooled [28]. These results were a clear indication that swelling and gelatinisation of starch granules were prevented by citrate substitution. Since the citrate starches were unable to form a gel, the study of textural properties was not carried out for these samples. 

The citrate starches did not form a gel and were therefore not subjected to texture analysis, whereas texture analysis was conducted on the native starches. Table 7 represents the textural parameters, including hardness, cohesiveness, and gumminess of native starches. There was a significant difference (*p* < 0.05) in the hardness among the native starches. Native chickpea had the highest hardness (39.06 ± 1.47 g), followed by native quinoa and cassava starches. There was no significant difference between the cohesiveness of the native cassava and chickpea starches, while that of native quinoa was significantly lower (*p* < 0.05). There was no significant difference between the gumminess of native cassava and quinoa starches. However, the gumminess of native chickpea starch was significantly higher. In summary, the inherent variations among native starches, such as variances in chemical composition, sizes of starch granules, amylose and amylopectin content, and molecular conformation, are the primary factors contributing to the diverse textural properties observed [18].

### 3.11. Polarised Light Microscopy Results

**Figure 8:**
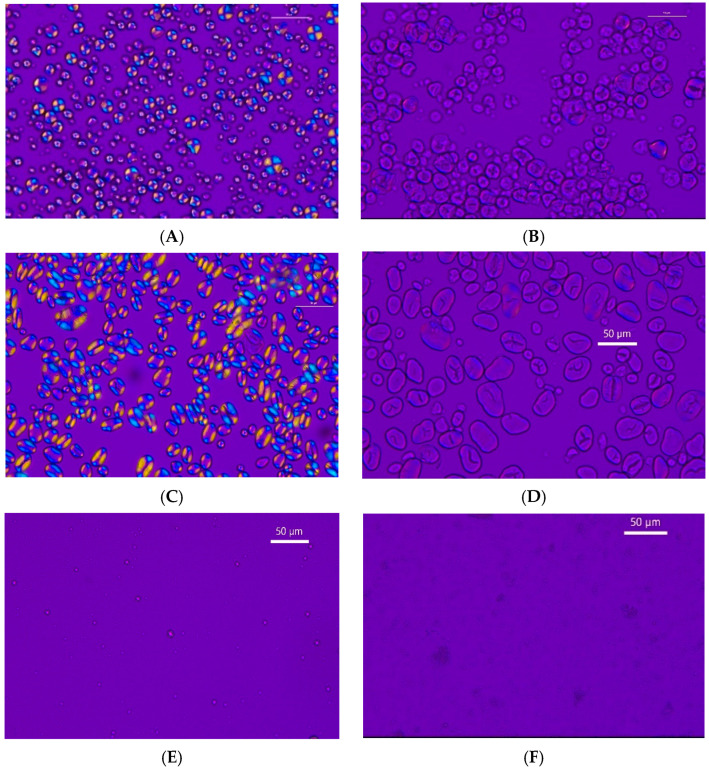
Polarised light microscopy of the native and citrate starches. Bars on the micrographs = 50 μm. (**A**): Native cassava starch; (**B**): Citrate cassava starch; (**C**): Native chickpea starch; (**D**): Citrate chickpea starch; (**E**): Native quinoa starch; (**F**): Citrate quinoa starch.

The results of polarised light microscopy are presented in Figure 8. Both native cassava and chickpea starches had a spherical-to-oval shape with smooth surfaces, while quinoa starch appeared as extremely tiny spheres. The granule diameters of native cassava, chickpea, and quinoa are 9–20 µm, 10–30 µm, and 0.766–4.050 µm, respectively [6,32,33]. This suggests that quinoa granules are the smallest in size, while chickpea granules are the largest among the three starches. This is in line with the particle size distribution results (Table 4). All three native starches had birefringence and Maltese crosses and showed no agglomerations. 

**Figure 9:**
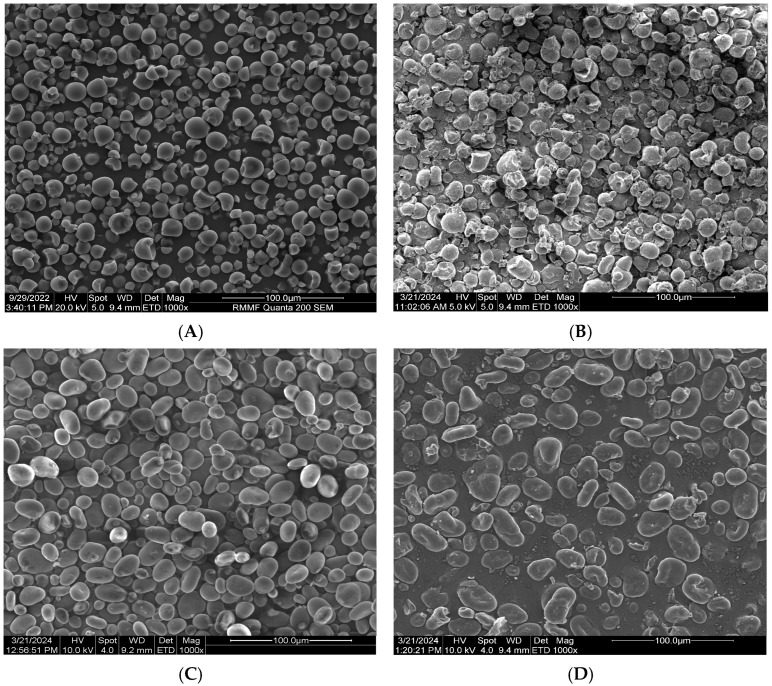

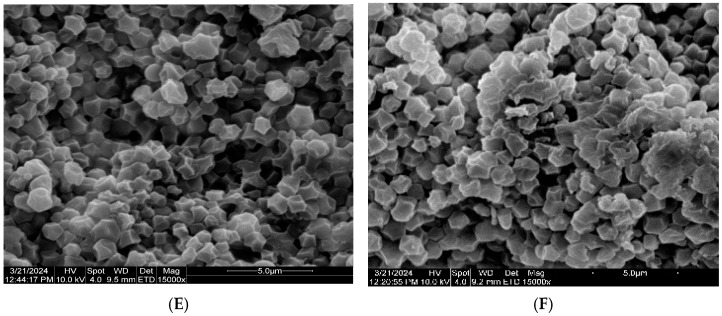
Electron microscopy images of the native and citrate starches. (**A**): Native cassava starch; (**B**): Citrate cassava starch; (**C**): Native chickpea starch; (**D**): Citrate chickpea starch; (**E**): Native quinoa starch; (**F**): Citrate quinoa starch.

However, the citrate starches were still in the granular form but lost their birefringence, which is an indication of the loss of molecular order and integrity due to the modification. The granules showed irregular and disrupted structures after modification. When the images of citrate starches were compared with their native counterparts, it was clear that the citrate starch granules have increased in size, had some agglomerations, and they display lower birefringence and a lesser number of Maltese crosses. Using the maximum magnification available (40× lens), quinoa starch granules were relatively smaller in size than the other starches and it was difficult to observe any clear differences between the shape of the citrate quinoa and its native counterpart. However, more detailed information was obtained using SEM (Figure 9). The same observations have been reported for citrate cassava, bananas, lentils, and sweet potatoes when observed under the scanning electron microscope [8]. These observations are due to the attack of the citric acid on the starch granule before entering into the inner area of the granule. When the crystallinity of the starch granules is disrupted, partial swelling and loss of birefringence and Maltese crosses occur [34]. The increased surface area was due to the disrupted and irregular surfaces causing agglomerations. It is evident that the citric acid modification has disrupted the crystalline regions of starch, thereby increasing the amorphous nature of starch granules [11]. The absence of birefringence and Maltese crosses was in line with X-ray and DSC results showing the destruction of the crystalline structure of native starches after being subjected to citric acid (Table 3 and Figure 3).

### 3.12. SEM Results

As shown in Figure 9, citrate treatment could cause superficial erosion and increase the size of the granules, as observed clearly in citrate cassava and chickpea starches observed under 1000× magnification. However, due to the tiny size of the quinoa starch, these changes are not clear even when using a larger magnification (15,000×). Native quinoa starch appeared as polygonal and irregular shapes (E). After modification, the main change observed was the increase in granular agglomeration and there were slight changes on the surface of quinoa starch. These changes for the citrate starches can affect their physicochemical properties, including water uptake and pasting and gelling properties, as discussed before. Similar findings have been reported for citrate rice starch [35], citrate corn and sorghum starch [14], and for citrate tiger nut starch [30]. 

## 4. Conclusions

In this research, both common (cassava starch) and emerging starches (chickpea and quinoa starch) were dry-treated with citric acid, a green and not-hazardous cross-linking agent. Under similar modification conditions, these starches exhibited similar behaviour despite their intrinsic differences. Using 30% citric acid (starch dry basis) resulted in more than 90% dietary fibre in the tested samples. A new feature of citrate starch, not observed in native starch, is the reduced hydrophilicity during heating in water. Interestingly, the reduction in water affinity was highest for citrate quinoa starch, followed by chickpea starch, and then cassava starch. This indicates that underutilised quinoa starch and emerging chickpea starch respond well to citrate modification to expand their food and non-food applications. Using 30% citric acid for modification resulted in a citrate starch that remained as a thin slurry during heating, was not gelatinised, showed no pasting viscosity, and exhibited no retrogradation during cooling. 

This unique characteristic will make citrate starch a suitable source of thermostable dietary fibre that can be included in a wide range of food systems as a rich source of fibre without impacting the physical properties (e.g., viscosity, texture) of final food products. However, citrate modification reduced the whiteness index and increased the yellowness of the treated starches, which should be considered for commercialisation and may necessitate additional bleaching. Further studies are required to enhance the food applications of citrate starches and to replace chemically cross-linked starches produced with high-risk cross-linking agents, which is critical for clean and green food labelling.

## Figures and Tables

**Table 1:** Degree of substitution (DS) and total dietary fibre (TDF) of the native and citrate starches.

Starch	DS (%)	TDF (%)
Native cassava	ND *	2.39 ± 0.01 ^d^
Citrate cassava	0.124 ± 0.011 ^a^	90.35 ± 0.20 ^b^
Native chickpea	ND	2.73 ± 0.13 ^d^
Citrate chickpea	0.117 ± 0.008 ^a^	95.53 ± 0.18 ^a^
Native quinoa	ND	6.70 ± 0.50 ^c^
Citrate quinoa	0.112 ± 0.007 ^a^	90.70 ± 0.50 ^b^

Mean ± standard deviation of triplicates. Values with different superscripts in a column are significantly different (*p* < 0.05). * ND: Not determined.

**Table 2:** Colour parameters of the native and citrate starches.

Starch	Image	Lightness (L*)	Redness (a*)	Yellowness (b*)	ΔE	WI
Native cassava	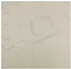	94.12 ± 1.31 ^a^	0.12 ± 0.04 ^a^	2.96 ± 0.03 ^e^	ND *	93.40 ± 1.17 ^a^
Citrate cassava	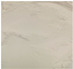	93.04 ± 0.87 ^a^	−0.20 ± 0.03 ^b^	5.47 ± 0.02 ^c^	2.89 ± 0.54 ^c^	91.14 ± 0.69 ^b^
Native chickpea	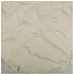	94.09 ± 0.29 ^a^	−0.52 ± 0.03 ^c^	2.60 ± 0.05 ^f^	ND	93.52 ± 0.25 ^a^
Citrate chickpea	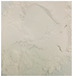	93.67 ± 1.47 ^a^	−1.00 ± 0.01 ^d^	7.07 ± 0.15 ^b^	4.62 ± 0.11 ^b^	90.41 ± 0.89 ^c^
Native quinoa	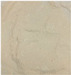	93.89 ± 0.43 ^a^	−0.09 ± 0.03 ^b^	4.73 ± 0.08 ^d^	ND	92.27 ± 0.37 ^b^
Citrate quinoa	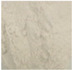	88.82 ± 0.84 ^b^	−0.13 ± 0.05 ^b^	11.41 ± 0.27 ^a^	8.40 ± 0.75 ^a^	84.02 ± 0.73 ^d^

Mean ± standard deviation of triplicates. Values with different superscripts in a column are significantly different (*p* < 0.05). * ND: Not determined. ΔE: Total colour change of citrate starches with respect to their native counterparts, WI: Whiteness index.

**Table 3:** Gelatinisation parameters of native and citrate starches obtained from DSC thermograms.

Starch	Gelatinisation Parameters			
	T_0_ (°C)	T_p_ (°C)	T_c_ (°C)	∆H (J/g)
Native cassava	65.03 ± 0.16 ^a^	70.75 ± 0.22 ^a^	77.41 ± 0.51 ^a^	10.82 ± 0.02 ^a^
Citrate cassava	* ND	ND	ND	ND
Native chickpea	61.60 ± 0.18 ^b^	65.89 ± 0.09 ^b^	68.05 ± 0.16 ^b^	4.91 ± 0.23 ^b^
Citrate chickpea	ND	ND	ND	ND
Native quinoa	57.50 ± 0.19 ^c^	62.17 ± 0.01 ^c^	65.61 ± 0.41 ^c^	4.99 ± 0.36 ^b^
Citrate quinoa	ND	ND	ND	ND

Mean ± standard deviation of two replicates. Values with different superscripts in the same column are significantly different (*p* < 0.05); * ND = Not detected, T_0_ = onset temperature, T_p_ = peak temperature, T_c_ = conclusion temperature, ∆H = gelatinisation enthalpy.

**Table 4:** The swelling power and the solubility of native and citrate starches.

Starch	Swelling Power (g/g)	Reduction in Swelling Power as Compared to the Native Starch (%)	Solubility (%)	Reduction in Solubility as Compared to the Native Starch (%)
Native cassava	7.60 ± 0.29 ^c^	-	5.97 ± 0.81 ^b^	-
Citrate cassava	4.91 ± 0.58 ^e^	−35.39	3.33 ± 1.16 ^c^	−44.22
Native chickpea	9.49 ± 0.67 ^b^	-	10.83 ± 3.79 ^a^	-
Citrate chickpea	3.32 ± 0.76 ^f^	−65.01	4.67 ± 2.08 ^c^	−56.87
Native quinoa	15.16 ± 0.86 ^a^	-	9.33 ± 0.29 ^a^	-
Citrate quinoa	4.95 ± 0.54 ^d^	−67.34	1.67 ± 1.16 ^c^	−82.10

Mean ± standard deviation of triplicates. Values with different superscripts in a column are significantly different (*p* < 0.05).

**Table 5:** Particle size distribution of native and citrate starches.

Sample	D10 (µm)	D50 (µm)	D90 (µm)	D (4,3) (µm)	D (3,2) (µm)	Span (µm)
Native cassava	9.22 ± 0.04 ^e^	14.10 ±0.06 ^e^	21.20 ± 0.17 ^e^	14.78 ± 0.07 ^d^	13.38 ± 0.07 ^d^	0.85 ± 0.01 ^d^
Citrate cassava	10.80 ± 0.00 ^c^	20.50 ± 0.16 ^b^	37.92 ± 0.57 ^b^	24.84 ± 0.51 ^a^	18.22 ± 0.13 ^b^	1.32 ± 0.02 ^b^
Native chickpea	12.76 ± 0.05 ^b^	19.02 ± 0.04 ^d^	28.42 ± 0.16 ^d^	19.90 ± 0.0 °C	18.10 ± 0.00 ^b^	0.82 ± 0.01 ^e^
Citrate chickpea	13.52 ± 0.16 ^a^	23.84 ± 0.05 ^a^	38.48 ± 0.11 ^a^	24.94 ± 0.05 ^a^	20.66 ± 0.13 ^a^	1.04 ± 0.02 ^d^
Native quinoa	3.81 ± 0.13 ^f^	10.58 ± 0.17 ^f^	19.76 ± 0.19 ^f^	12.34 ± 0.54 ^e^	11.81 ± 0.98 ^e^	1.50 ± 0.02 ^a^
Citrate quinoa	10.62 ± 0.04 ^d^	19.86 ± 0.09 ^c^	35.04 ± 0.23 ^c^	21.62 ± 0.13 ^b^	17.62 ± 0.04 ^c^	1.23 ± 0.01 ^c^

Mean ± standard deviation of five replicates. Values with different superscripts in the same column are significantly different (*p* < 0.05). D10, particle diameter corresponding to 10% cumulative undersize; D50, median particle size distribution; D90, particle diameter corresponding to 90% cumulative undersize; D (4,3), volume-weighted mean diameter, D (3,2); surface-weighted mean diameter.

**Table 6:** Pasting parameters of native and citrate starches extracted from the RVA graphs.

Starch	PV (cP)	TV (cP)	BV (cP)	FV (cP)	SV (cP)
Native cassava	3532.00 ± 43.84 ^a^	1545.50 ± 23.33 ^c^	1986.50 ± 20.51 ^a^	2633.50 ± 23.33 ^c^	1088.00 ± 28.8 ^b^
Citrate cassava	ND *	ND	ND	ND	ND
Native chickpea	2291.00 ± 1.41 ^c^	1806.00 ± 59.40 ^b^	485.00 ± 60.81 ^b^	3682.00 ± 1.41 ^a^	1876.00 ± 60.81 ^a^
Citrate chickpea	ND	ND	ND	ND	ND
Native quinoa	2500.50 ± 45.96 ^b^	2152.00 ± 49.50 ^a^	358.50 ± 10.61 ^c^	2992.50 ± 53.03 ^b^	840.50 ± 3.54 ^c^
Citrate quinoa	ND	ND	ND	ND	ND

PV = Pasting viscosity; TV = Trough viscosity; BV = Breakdown viscosity; FV = Final viscosity; SV = Setback viscosity, * ND = Not detected. Different superscript letters in each column show significant statistical differences (*p* < 0.05).

**Table 7:** Textural properties of the native starches.

Starch	Hardness (g)	Cohesiveness	Gumminess (g)
Native cassava	26.67 ± 0.83 ^c^	1.24 ± 0.46 ^a^	32.97 ± 1.13 ^b^
Native chickpea	39.06 ± 1.47 ^a^	1.14 ± 0.01 ^a^	35.09 ± 1.81 ^a^
Native quinoa	29.90 ± 0.22 ^b^	1.04 ± 0.02 ^b^	30.94 ± 0.47 ^c^

Values are the standard deviation averages of triplicates. Different superscripts in each column show significant statistical differences (*p* < 0.05).

## Data Availability

The original contributions presented in this study are included in the article. Further inquiries can be directed to the corresponding authors.

## References

[1] Bojarczuk A., Skąpska S., Mousavi Khaneghah A., Marszałek K. (2022). Health benefits of resistant starch: A review of the literature. J. Funct. Foods.

[2] Veronese N., Solmi M., Caruso M.G., Giannelli G., Osella A.R., Evangelou E., Maggi S., Fontana L., Stubbs B., Tzoulaki I. (2018). Dietary fiber and health outcomes: An umbrella review of systematic reviews and meta-analyses. Am. J. Clin. Nutr..

[3] Niu H., Zhao F., Ji W., Ma L., Lu B., Yuan Y., Yue T. (2024). Structural, physicochemical properties and noodle-making potential of quinoa starch and type 3, type 4, and type 5 quinoa resistant starch. Int. J. Biol. Macromol..

[4] Wang Z., Wang S., Xu Q., Kong Q., Li F., Lu L., Xu Y., Wei Y. (2023). Synthesis and Functions of Resistant Starch. Adv. Nutr..

[5] Punia Bangar S., Sunooj K.V., Navaf M., Phimolsiripol Y., Whiteside W.S. (2024). Recent advancements in cross-linked starches for food applications—A review. Int. J. Food Prop..

[6] Mhaske P., Wang Z., Farahnaky A., Kasapis S., Majzoobi M. (2022). Green and clean modification of cassava starch—Effects on composition, structure, properties and digestibility. Crit. Rev. Food Sci. Nutr..

[7] Reddy N., Yang Y. (2010). Citric acid cross-linking of starch films. Food Chem..

[8] Remya R., Jyothi A.N., Sreekumar J. (2018). Effect of chemical modification with citric acid on the physicochemical properties and resistant starch formation in different starches. Carbohydr. Polym..

[9] Shen L., Xu H., Kong L., Yang Y. (2015). Non-toxic crosslinking of starch using polycarboxylic acids: Kinetic study and quantitative correlation of mechanical properties and crosslinking degrees. J. Polym. Environ..

[10] Wang C., Fang S., Ren C., Huang C., Zhu H., Zhang X., Zhao J. (2023). Cross-linked modifications of starches from colored highland barley and their characterizations, digestibility, and lipolysis inhibitory abilities in vitro. Food Res. Int..

[11] Kumar Y., Singh S., Saxena D.C. (2022). Controlling the properties of starch from rice broken by crosslinking with citric acid and sodium trimetaphosphate. Starch-Stärke.

[12] Adhiyamaan P.S., Parimalavalli R. (2020). Effect of dual modification on crystalline formation of resistant starch from cassava. J. Food Meas. Charact..

[13] Mei J.-Q., Zhou D.-N., Jin Z.-Y., Xu X.-M., Chen H.-Q. (2015). Effects of citric acid esterification on digestibility, structural and physicochemical properties of cassava starch. Food Chem..

[14] Shaikh F., Ali T.M., Mustafa G., Hasnain A. (2019). Comparative study on effects of citric and lactic acid treatment on morphological, functional, resistant starch fraction and glycemic index of corn and sorghum starches. Int. J. Biol. Macromol..

[15] Liang W., Zhang Q., Guo S., Ge X., Shen H., Zeng J., Gao H., Li W. (2024). Investigating the influence of CaCl_2_ induced surface gelatinization of red adzuki bean starch on its citric acid esterification modification: Structure–property related mechanism. Food Chem..

[16] Li G., Zhu F. (2018). Quinoa starch: Structure, properties, and applications. Carbohydr. Polym..

[17] Bashir M., Haripriya S. (2016). Physicochemical and structural evaluation of alkali extracted chickpea starch as affected by γ-irradiation. Int. J. Biol. Macromol..

[18] Jan K.N., Panesar P.S., Singh S. (2017). Process standardization for isolation of quinoa starch and its characterization in comparison with other starches. J. Food Meas. Charact..

[19] Mao L., Mhaske P., Farahnaky A., Majzoobi M. (2023). Effect of dry heating on some physicochemical properties of protein-coated high amylose and waxy corn starch. Foods.

[20] Ghosh Dastidar T., Netravali A.N. (2012). ‘Green’ crosslinking of native starches with malonic acid and their properties. Carbohydr. Polym..

[21] Golachowski A., Drożdż W., Golachowska M., Kapelko-Żeberska M., Raszewski B. (2020). Production and Properties of Starch Citrates—Current Research. Foods.

[22] He M., Chen J., Liu W., Lin J. (2024). Solvent-free Synthesis of Starch Citrate Via Dry Heat Reaction: An In-depth Analysis on Structural and Physicochemical Properties. LWT.

[23] Alimi B.A., Workneh T.S. (2018). Structural and physicochemical properties of heat moisture treated and citric acid modified acha and iburu starches. Food Hydrocoll..

[24] Apriyanto A., Compart J., Fettke J. (2022). A review of starch, a unique biopolymer—Structure, metabolism and in planta modifications. Plant Sci..

[25] Dong H., Vasanthan T. (2020). Effect of phosphorylation techniques on structural, thermal, and pasting properties of pulse starches in comparison with corn starch. Food Hydrocoll..

[26] Li G., Xu X., Zhu F. (2019). Physicochemical properties of dodecenyl succinic anhydride (DDSA) modified quinoa starch. Food Chem..

[27] Junejo S.A., Wang J., Liu Y., Jia R., Zhou Y., Li S. (2022). Multi-scale structures and functional properties of quinoa starch extracted by alkali, wet-milling, and enzymatic methods. Foods.

[28] Karma V., Gupta A.D., Yadav D.K., Singh A.A., Verma M., Singh H. (2022). Recent developments in starch modification by organic acids: A review. Starch-Stärke.

[29] Egharevba H.O. (2019). Chemical properties of starch and its application in the food industry. Chemical Properties of Starch.

[30] Lv X., Guo C., Ma Y., Liu B. (2022). Effect of citric acid esterification on the structure and physicochemical properties of tigernut starch. Int. J. Biol. Macromol..

[31] Wei W., Wu M., Ren W., Yu H., Sun D. (2024). Preparation of crosslinked starches with enhanced and tunable gel properties by the cooperative crosslinking-extrusion combined modification. Carbohydr. Polym..

[32] Rayner M., Timgren A., Sjöö M., Dejmek P. (2012). Quinoa starch granules: A candidate for stabilising food-grade Pickering emulsions. J. Sci. Food Agric..

[33] Wang Z., Mhaske P., Farahnaky A., Kasapis S., Majzoobi M. (2022). Cassava starch: Chemical modification and its impact on functional properties and digestibility, a review. Food Hydrocoll..

[34] Cornejo Y., Martínez-Cruz O., Toro-Sánchez C., Wong Corral F., Borboa-Flores J., Cinco-Moroyoqui F. (2018). The structural characteristics of starches and their functional properties. CyTA-J. Food.

[35] Zhong C., Xiong Y., Lu H., Luo S., Wu J., Ye J., Liu W. (2021). Preparation and characterization of rice starch citrates by superheated steam: A new strategy of producing resistant starch. LWT-Food Sci. Technol..

